# KAT5 Inhibitor NU9056 Suppresses Anaplastic Thyroid Carcinoma Progression through c-Myc/miR-202 Pathway

**DOI:** 10.1155/2022/2014568

**Published:** 2022-02-11

**Authors:** Wenjing Xu, Liwei Xie, Yingying Yang, Jiayu Xu, Shang Cai, Ye Tian

**Affiliations:** ^1^Department of Radiotherapy and Oncology, The Second Affiliated Hospital of Soochow University, Suzhou 215004, China; ^2^Institute of Radiotherapy and Oncology, Soochow University, Suzhou 215004, China; ^3^Suzhou Key Laboratory for Radiation Oncology, Suzhou 215004, China

## Abstract

**Background:**

Anaplastic thyroid carcinoma (ATC) is considered to be one of the most aggressive cancers. Our previous study proved that highly expressed lysine acetyltransferase 5 (KAT5) in ATC is associated with a poorer prognosis. Here, this study examined the effects of a KAT5 inhibitor (NU9056) in human ATC cells.

**Methods:**

First, the *Cancer* Genome Atlas (TCGA) dataset was used to detect the relationship between KAT5 expression and outcomes of thyroid carcinoma patients. Then, both *in vitro* and *in vivo* experiments were conducted to investigate the effects of NU9056 on normal and ATC human thyroid cells. Finally, microRNA sequencing, qPCR, and dual-luciferase reporter assay were performed to explore potential mechanisms by identifying downstream microRNA related to NU9056.

**Results:**

KAT5 dysregulation correlated with more advanced-stage and poorer outcomes of thyroid carcinoma patients. Endogenous KAT5 protein and mRNA levels were much higher in ATC cells than in normal thyroid cells. Suppression of KAT5 by NU9056 inhibited survival, growth, migration, invasion, and tube formation, and increased radiosensitivity and chemosensitivity in ATC cells but showed no impact on normal thyroid cells. Mechanistically, microRNA-202-5p (miR-202) was identified as the most significantly decreased miRNA after NU9056 treatment. Knockdown of miR-202 suppressed ATC cell progression, while forced expression of miR-202 partially blocked the inhibitory effect of NU9056 on ATC cells. Furthermore, c-Myc was validated as the transcription factor of miR-202, and NU9056 decreased the c-Myc protein level by shortening its half-life. Finally, we proved that NU9056 inhibited ATC proliferation in vivo.

**Conclusions:**

Our results indicated that NU9056 targets KAT5, shortens c-Myc half-life, subsequently downregulates miR-202 expression, and results in the suppression of ATC cells. Overall, KAT5 could be a potential target for clinical treatment for ATC.

## 1. Introduction

Anaplastic thyroid carcinoma (ATC) is highly malignant due to its proliferative capacity and invasive nature [1,2]. The prognosis of ATC patients still remains extremely poor, with a median survival of only around 5–6 months [1,2]. Thus, in spite of its rarity, ATC accounts for around half of thyroid cancer-related death [1,2]. Upon diagnosis, more than half of ATC patients already present with local invasion and distant metastasis, and only in 10% of cases the disease is confined to the thyroid gland [1,2]. Therefore, to change this dismal situation it is vital to understand the molecular mechanism that drives ATC progression.

Although there have been several suggestions as to mechanisms driving ATC progression, treatment with agents that target these mechanisms does not improve the prognosis of ATC patients [3–5]. Since ATC is uncommon, making most studies is harder to recruit a sufficient number of participants to draw conclusions [2].

Based on 82 ATC clinical samples, our previous study found a strong correlation of lysine acetyltransferase 5 (KAT5, also known as Tip60) overexpression with poorer prognosis and tumor metastasis of ATC [6]. KAT5 belongs to the MYST family of acetyltransferases [7], and its substrate includes both histone proteins (histone H4 and H2A) and nonhistone proteins (ATM, p53, p21, estrogen receptor, and so on) [7]. KAT5 is, therefore, involved in regulating many important physiological processes. Recently, increasing evidence showed that KAT5 also functions as activators of oncogenes in various types of cancers. Wang et al. found that KAT5-regulated SPZ1-TWIST1 complex leads to invasion and metastasis of liver cancer [8]. Meanwhile, Li et al. proved that KAT5-mediated SMAD3 acetylation promotes melanoma progression and invasion [9]. We also showed that KAT5 acetylates and stabilizes c-Myc protein by blocking the proteasome system [6]. The Myc proteins are the products that are encoded by the well-known *MYC* family of proto-oncogenes [10]. Among them, transcription factor c-Myc constitutes the central transcriptional regulator network that regulates the expression of around one-sixth of all human genes and plays key roles in several cellular processes including metabolism, proliferation, differentiation, apoptosis, and so on [11]. Expression of c-Myc is enhanced and deregulated in many human tumors [11], suggesting a possibility that highly expressed KAT5 in ATC contributes to the progression of tumor by modulating c-Myc. These findings also suggest that aberrant high expression of KAT5 may drive ATC progression and, therefore, that targeting KAT5 could provide a novel and efficient clinical strategy against ATC.

NU9056 is a cell-permeable disulfane compound that is a potent and highly selective KAT5 acetyltransferase inhibitor [12]. NU9056 displays favorable physicochemical and pharmacokinetic properties and a good safety profile, which makes it suitable for potential clinical development.

MicroRNA (miRNA) is a type of short, single-stranded, noncoding RNA widely expressed in mammalian cells. It regulates cell gene expression via blocking mRNA translation or causing mRNA decay [13]. At present, there is a lot of evidence to show that abnormally regulated miRNAs contribute to the occurrence, development, and treatment resistance of several malignant tumors, including thyroid carcinoma, lung cancer, colorectal cancer, and others [14]. It is plausible that in ATC, miRNA is regulated by KAT5 and contributes to tumor progression. However, KAT5-mediated regulation of miRNA in ATC has not yet been investigated.

Since KAT5 represents a novel potential oncotarget in ATC treatment, we focused on investigating the potential antitumor activity of NU9056 in human ATC cells and the underlying mechanisms through miRNA sequencing. In this study, we found that NU9056 blocks KAT5 activity leading to an antitumoral effect. The underlying mechanism is based on c-Myc blockage and consequent miR-202 downregulation. Altogether, our data suggested that KAT5 could be a potential target for clinical treatment for ATC.

## 2. Materials and Methods

### 2.1. Cell Culture and Transfection

Human ATC cell CAL-62 and embryonic kidney cell 293T were purchased from the Cell Bank of Chinese Academy of Science (Shanghai, China). Human umbilical vein endothelial cells (HUVECs) were purchased from Gefen Corp (Shanghai, China). Human ATC cell line 8505C was generously provided by Dr. Xi Wei (Tianjin Medical University *Cancer* Institute and Hospital, China). Human normal thyroid cell Nthy-ori 3–1 was purchased from Antonio Corp (Suzhou, China). All the cells were cultured in DMEM medium supplemented with 10% FBS and 1% penicillin and streptomycin, in a humidified incubator at 37 °C with 5% CO2.

For the cell transfection, vector, or KAT5 plasmid, con-shRNA, or KAT5-shRNA were designed and synthesized by GeneChem Corp (Shanghai, China). Transfection was accomplished using Lipofectamine 2000 (Invitrogen) according to the manufacturer's instruction: ATC cells were seeded into 12-well plates (2 × 10^5^ cells/well). Then, 30 pmol of con-shRNA or KAT5-shRNA, or 3ug of vector or KAT5 plasmid was transfected. After 48 h, KAT5 expression was determined by Western blot (WB) and qPCR.

### 2.2. RT-qPCR

Total RNAs were extracted from cells by TRIzol LS Reagent (Invitrogen) according to manufacturer's instruction, and 2 µg RNA was used for reverse transcription. Thermo Scientific cDNA Reverse Transcription Kit (#K1621) was used, and the steps and conditions were performed according to the manufacturer's instructions: (1) 25°C for 10 min, (2) 37°C for 120 min, and then (3) 85°C for 5 min. The cDNA was next amplified by using SYBR Green Master Mix as follows: 50°C for 2 min, 95 °C for 10 min, and then 40 cycles at 95°C followed by 60°C for 1 min. The primer sequences were *KAT5* forward primer: 5′-AATGTGGCCTGCATCCTAAC-3′; *KAT5* reverse primer: 5′-TGTTTTCCCTTCCACTTTGG-3′; *GAPDH* forward primer: 5′-CGACCACTTTGTCAAGCTCA-3′; *GAPDH* reverse primer: 5′-AGGGGTCTACATGGCAACTG-3′.

For the quantification of miRNA, miRNA cDNA Synthesis Kit and SYBR Green miRNA qPCR Assay Kit were used (CWBio, Beijing). Each step was conducted according to the manufacturer's instructions. Briefly, in the reverse transcription process, 2 µg of total RNA was mixed with 3 µl of RT primer (provided in the kit) and other reagents, incubated at 42°C for 50 min, and then further incubated at 85°C for 5 min. The cDNA was then used as a template for exponential amplification using the SYBR Green miRNA qPCR Assay Kit as follows: 95°C for 10 min, 40 cycles at 95°C for 10 s, and followed by 60°C for 1 min. The primer sequences were U6 forward primer: 5′-ATTGGAACGATACAGAGAAGATT-3′, U6 reverse primer: 5′-GGAACGCTTCACGAATTTG-3′, hsa-miR-202-5p forward primer: 5′- CCGCTCGTTCCTATGCATATAC-3′, and hsa-miR-202-5p reverse primer: 5′- CAGAGCAGGGTCCGAGGTA-3′.

### 2.3. Western Blotting

As previously described [6], after extraction, the quantified protein lysates were separated using SDS gels, transferred to PVDF blots, and blocked using skim milk. Membranes were then incubated overnight with primary antibodies at 4°C, washed, and incubated with HRP-conjugated secondary antibodies for another 2 h at room temperature. Next, the enhanced chemiluminescence reagents were added for visualization of the target protein bands. The primary antibodies used were as follows: KAT5 (Abcam, in a 1 : 2000 dilution), lamin A/C (Cell Signaling Technology, in a 1 : 5000 dilution), c-Myc (Santa Cruz, in a 1 : 2000 dilution), acetyl-histone H2A (CST, in a 1 : 2000 dilution), total histone H2A (Abcam, in a 1 : 2000 dilution), acetyl-histone H4 (CST, in a 1 : 2000 dilution), total histone H4 (Abcam, in a 1 : 2000 dilution), and *β*-actin(Abcam, in a 1 : 5000 dilution). Secondary antibodies used were as follows: anti-mouse IgG (Abcam, in a 1 : 10000 dilution) and anti-rabbit IgG (Abcam, in a 1 : 10000 dilution).

### 2.4. NU9056

#### 2.4.1. NU9056 Was Provided by Absin (Shanghai, China)

As for the concentration, according to a previous study [12], in growth inhibition assay, the concentration required to inhibit cell growth by 50% of NU9056 for human prostate cancer cells was about 24 uM. Therefore, in the pre-experiment NU9056 concentrations were chosen from 1 uM to 100 uM. Finally, in the normal experiment NU9056 concentrations were chosen from 2.5 uM to 40 Um.

To maintain drug concentration during the colony formation assay, mediums containing doses of NU9056 were changed every 3 days.

### 2.5. Cell Viability

Cells were plated into 96-well plates (4 × 10^3^ cells/well), cultured overnight, and treated with NU9056 for the indicated time. Cell counting kit-8 (CCK-8) was purchased from Beyotime. Then, CCK-8 solution (10 ul/well) was added and incubated for 2 h at 37 °C. Next, the optical density (OD) value was measured using a microplate reader at a wavelength of 450 nm.

### 2.6. Transwell Migration and Invasion Assay

As described previously [6], cells cultured without FBS were seeded to the upper chamber of each Transwell with (invasion) or without (migration) Corning Matrigel matrix (diluted by 1 : 8), and a culture medium containing 10% FBS was added to the lower chambers for chemoattracion. Then, cells on the bottom were fixed by 4% paraformaldehyde, dyed by violet crystal, and then pictured and counted.

### 2.7. Colony Formation Assay

Cells were seeded into 6-well tissue culture plates and incubated overnight (2000 cells/well). Then, NU9056 was added at indicated doses and continued to incubate for around 2 weeks. Furthermore, the colonies were washed, fixed, stained crystal violet, and counted. Colonies consisting of 50 or more cells were counted.

### 2.8. Chemosensitivity Experiment

According to our previous unpublished data, the half-maximal inhibitory concentration of cisplatin for human thyroid cancer cells for the CCK-8 assay was around 1 µg/ml. Therefore, we applied cisplatin concentrations ranging from 0.1 to 3 µg/ml. NU9056 was added with a concentration of 10 µM. Cells were then plated into 96-well plates at a density of 4 × 10^3^ cells/well and were subsequently treated with the indicated doses of cisplatin for 48 h. Following this, 10 µl of CCK-8 solution was added to each well and incubated for 2 h at 37°C. Next, the OD value was measured using a microplate reader at a wavelength of 450 nm.

According to our previous unpublished data, for the transwell migration assay (described above), 0.5 µg/ml of cisplatin caused no apparent inhibitory effect on the viability of human thyroid cancer cells and was chosen for further study.

### 2.9. Radiosensitivity Experiment

Cells were seeded into a 6-well plate (the cell numbers were as follows: 400 for 0 Gy, 600 for 1 Gy, 800 for 2 Gy, 1200 for 3 Gy, and 1600 for 4 Gy), and NU9056 was added at the indicated doses the next day. Meanwhile, cells were irradiated and incubated for another 14 days. Following this, the cells were fixed and dyed with crystal violet. Then, colonies consisting of 50 or more cells were counted.

### 2.10. Immunofluorescence

As previously described [15], cells were seeded into a 6-well plate (1.5 × 10^5^ cells/well) over a glass slide. At the corresponding time points after NU9056 treatment, cells were flushed with ice-cold PBS and fixed with 4% paraformaldehyde. Immunofluorescence for c-Myc (Santa Cruz, 1 : 400) was processed according to the previously published protocol. The next day, the glass slides were washed and the secondary antibody was incubated (anti-mouse IgG (*H* + *L*)F(ab')2 Fragment (#4408), Cell Signaling Technology, 1 : 500). Then, cells were pictured under an inverted fluorescence microscope (Olympus). Finally, the fluorescence intensity was analyzed by Image J.

### 2.11. Tube Formation Assay

ATC or normal thyroid cells were treated with indicated doses of NU9056. After 24 h, 1 × 10^4^ HUVEC cells and 1 × 10^4^ ATC or normal thyroid cells were seeded to the 96-well plate that were coated with Corning Matrigel matrix (in a 1 : 8 dilution with DMEM), and each dose exposure of NU9056 was replicated 4 times. After coculture for 24 h, the tube structures were pictured by Olympus inverted microscope (X 100). Then, the lengths of tubes were counted by Image J.

### 2.12. Dual-Luciferase Reporter Assay

 PGL4-miR-202-5p-promoter (2000 bp; TSS −2000 to 0) and pcDNA3.1-c-Myc were designed and synthesized by GenePharma (Shanghai, China). 293T cells were seeded to a 24-well plate (2 × 10^5^ cells/well). Then, pcDNA3.1, PGL4+RL-TK, PGL4-miR-202-5p-promoter, or pcDNA3.1-c-Myc was transfected into the 293T cells using Lipofectamine 2000. The luciferase activity was then measured by a dual-luciferase reporter assay. Briefly, 100 µl of passive lysis buffer was added into each well and incubated for 15 min. Next, 20 µl of LARII reagent was added into each well, and the firefly luciferase was determined by a microplate reader. Then, 20ul Stop & Glo® Reagent was added into each well, and the Renilla luciferase was determined by a microplate reader, which was used as an internal control.

### 2.13. Oncomine Database

To investigate the levels of c-Myc expression in ATC and other types of thyroid carcinoma, the Oncomine database was searched (https://software.oncomine.com/). *Cancer* vs. cancer was chosen as the analysis type, and thyroid gland carcinoma was chosen for the cancer type. Next, a dataset called Giordano thyroid that contains 99 cases of both ATC and differentiated thyroid gland carcinoma was chosen. Finally, the expression levels of c-Myc in ATC and other types of differentiated thyroid carcinoma were obtained.

### 2.14. MiRNA Sequencing

The miRNA sequencing experiment and data analysis of 6 samples (human ATC cell 8505C transfected with vector or KAT5 plasmid, each group replicated in triplicate) were conducted by OE Biotechnology Co., Ltd. (Shanghai, China). One *μ*g total RNA of each sample was used for the small RNA library construction using TruSeq Small RNA Sample Prep Kits (Cat. No. RS-200-0012, Illumina, USA) following the manufacturer's recommendations. Briefly, total RNA was ligated with 3′ and 5′ adapters. Then, 6 µl 5′ and 3′ adapter-ligated RNA with 1 *μ*l RNA RT primer was mixed and incubated at 70°C for 2 min. Next, 5.5 *μ*l of the reverse transcription mix was added and incubated at 50°C for 1 h, and the tube was then placed on ice. Following this, PCR amplification was performed according to the manufacturer's protocol: 1 cycle at 98°C for 30 s, 15 cycles at 98°C for 10 s, 60°C for 30 s, 72°C for 30 s, 1 cycle at 72°C for 10 min, and then held at 4°C. Finally, the cDNA construct was purified and recovered. The libraries were sequenced using the Illumina HiSeq *X* Ten platform, and 150 bp paired-end reads were generated.

After high-throughput sequencing and subsequent quality control, high-quality clean reads were obtained for analysis. The number of clean reads of all samples ranged from 10, 290, 533 to 14, 625, 392. The length of all clean reads was between 20 and 24 nucleotides, with the largest population of reads being 23 nucleotides. Small RNAs were classified and annotated on the basis of the Rfam database, species reference transcript, and repeat sequence database. The known miRNAs were annotated using the miRBase v.21 database. Those with a *p* value < 0.05 were defined as differentially expressed miRNAs.

### 2.15. In Vivo Assay

The animal experimental protocol was approved by the ethics committee of Soochow University (ECSU-2019000200). Male BALB/*c* SPF nude mice (*n* = 20, 4–6 weeks old, weight 18–22 g) were purchased from the Soochow University. Injections of 5 × 10^6^ CAL-62 cells were subcutaneously administered into the right flank. Mice were then randomly allocated into either the control group or the NU9056 group (both *n* = 10). The NU9056 group received intraperitoneal injection of NU9056 diluted in saline with a concentration of 0.5 µg/µl every 2 days. The NU9056 injection dose was 200 µg/100 g. The tumor weights were then measured. The long and short diameters of tumors were measured using calipers every 3 days, and tumor volumes were calculated using the formula: volume = 0.52 × long diameter × (short diameter)^2^. After 21 days, mice were killed, and tumors were removed. Next, the total tumor RNA was extracted using the TRIzol method, and RT-qPCR was conducted for miRNA.

### 2.16. Statistics Analysis

Data were expressed as mean ± SD. Comparisons were conducted using unpaired Student's *t*-test. A *p* < 0.05 was considered to be statistically significant. Statistical analysis was performed using SPSS 22.0.

## 3. Results

### 3.1. Aberrantly Expressed KAT5 Correlated with More Advanced-Stage and Poor Prognosis in Thyroid Carcinoma and Was Overexpressed in ATC

**Figure 1:**
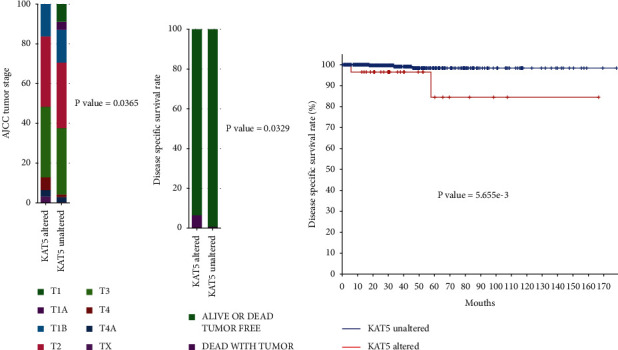
Elevated KAT5 expression correlates with poor survival in patients with thyroid carcinoma. TCGA database was used to analyze the correlation between KAT5 expression and T staging (A) or disease-specific survival rate (B, C) of patients with thyroid carcinoma.

In this study, we first assessed the expression levels of KAT5 in human thyroid carcinoma, and the TCGA database was downloaded. In a dataset that contains 500 papillary thyroid cancer patients (TCGA, PanCancer Atlas), we found that KAT5 alteration cases (mostly were KAT5 mRNA high expression) had more advanced *T* stages and poorer survival than KAT5 normally expressed ones (defined as KAT5 mRNA normal expression) (Figures 1(a)-1(c)).

### 3.2. NU9056 Specifically Inhibits Cell Viability and Proliferation in KAT5 Overexpressed Human ATC Cells

**Figure 2:**
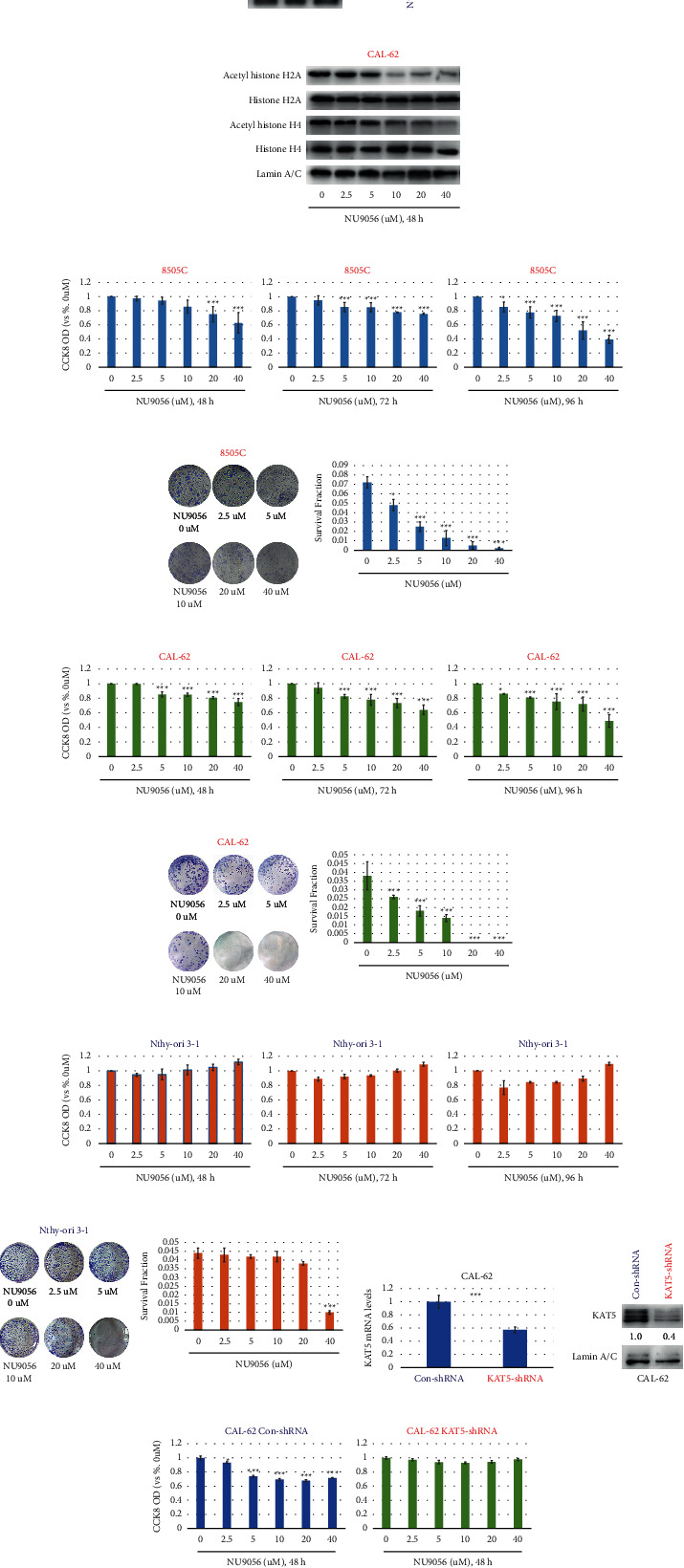
NU9056 inhibits survival and proliferation in human ATC cells. Endogenous KAT5 protein (a) and mRNA (B) expressions were detected by Western blot or qRT-PCR in human ATC (8505C and CAL-62) and normal thyroid (Nthy-ori 3–1) cells. Then, CAL-62 cells were left untreated or treated with NU9056 (2.5–40uM) for 48h. Then, the expression levels of acetyl-histone H2A, total histone H2A, acetyl-histone H4, and total histone H4 were detected by Western blot (C). Next, 8505C (D, E), CAL-62(F, G), and Nthy-ori 3–1(H, I) cells were left untreated or treated with NU9056 (2.5–40uM), cells were further cultured in indicated time periods, and then, cell viability (D) F, (H) and proliferation (E) G, (I) were tested by the appropriate assays. Cal-62 cells transiently transfected with either control or KAT5 shRNA. The knockdown effect was detected by RT-PCR (J) or Western blot analysis (K), cells were left untreated or treated with NU9056 (2.5–40uM), cells were further cultured in indicated time periods, and then, cell viability (L) was tested by the appropriate assay. ^*∗*^*p* < 0.05 vs. “Ctrl” group, ^*∗∗∗*^*p* < 0.01 vs. “Ctrl” group.

We then found that endogeneous KAT5 expressions were much higher in ATC cell lines (8505C and CAL-62) than in a normal thyroid cell line (Nthy-ori 3–1) at both protein and mRNA levels (Figures 2(a), 2(b)).

NU9056 is a highly selective KAT5 acetyltransferase inhibitor, in order to prove that NU9056 is effective in blocking KAT5 activity, and human ATC cells CAL-62 were left untreated or treated with NU9056 (2.5–40uM) for 48h. Then, the levels of KAT5's downstream, histone H2A, and histone H4 acetylation were detected by Western blot. The results showed that NU9056 decreased the expression levels of acetyl-histone H2A and acetyl-histone H4 in a concentration-dependent manner but had no effect on the expression levels of total histone H2A and total histone H4 (Figure 2(c)).

Next, we studied the effects of elevated KAT5 expression on ATC viability and proliferation by treating human ATC cells with NU9056 at a range of concentrations (2.5–40 µM). The results of the CCK8 assay (Figures 2(d), 2(f), and 2(h)) showed that NU9056 potently inhibited ATC cell viability in a concentration-dependent manner but had no inhibitory effect on normal thyroid cells. Similarly, in the colony formation assay (Figures 2(e), 2(g), and 2(i)), NU9056 potently inhibited ATC cell proliferation in a concentration-dependent manner but had a weakened or no inhibitory effect on normal thyroid cells. Furthermore, after con-shRNA or KAT5-shRNA was transfected into CAL-62 cells (Figures 2(j) and 2(k)), and the inhibitory effect of NU9056 on the viability of ATC cells was totally blocked after downregulation of KAT5 (Figure 2(l)).

### 3.3. NU9056 Specifically Inhibits Migration, Invasion, and Tube Formation in KAT5 Overexpressed Human ATC Cells

**Figure 3:**
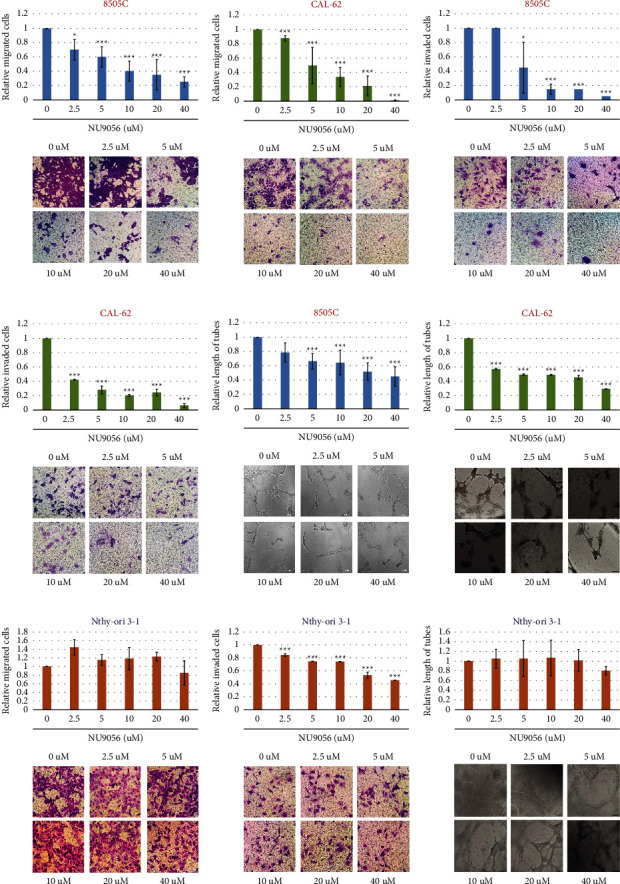
NU9056 inhibits migration, invasion, and tube formation in human ATC cells8505 C (A) C, (E) and CAL-62 (B) D, (F) cells were left untreated or treated with NU9056 (2.5–40uM), and then, cell migration (A, B), invasion (C, D), and tube formation ability (E, F) were tested by the appropriate assay. ^*∗*^*p* < 0.05 vs. “Ctrl” group, ^*∗∗∗*^*p* < 0.01 vs. “Ctrl” group.

Next, the effect of NU9056 on migration and invasion ability of ATC cells was evaluated by Transwell assay. We found that NU9056 significantly inhibited ATC cell migration and invasion in a dose-dependent way (Figures 3(a)-3(d)). Since the angiogenic activity is also vital for tumor invasion and metastasis, a tube formation assay was conducted. The results showed that NU9056 also remarkably inhibited tube formation of microvascular endothelial cells that were cocultured with ATC cells (Figures 3(e), 3(f)). Interestingly, compared with KAT5 overexpressed human ATC cell lines, NU9056 showed no or weakened inhibitory effect on migration, invasion, and tube formation ability of KAT5 low-expressed normal thyroid cells (Figures 3(g)–3(i)).

### 3.4. NU9056 Specifically Increases Radio- and Chemosensitivities of KAT5 Overexpressed Human ATC Cells

**Figure 4:**
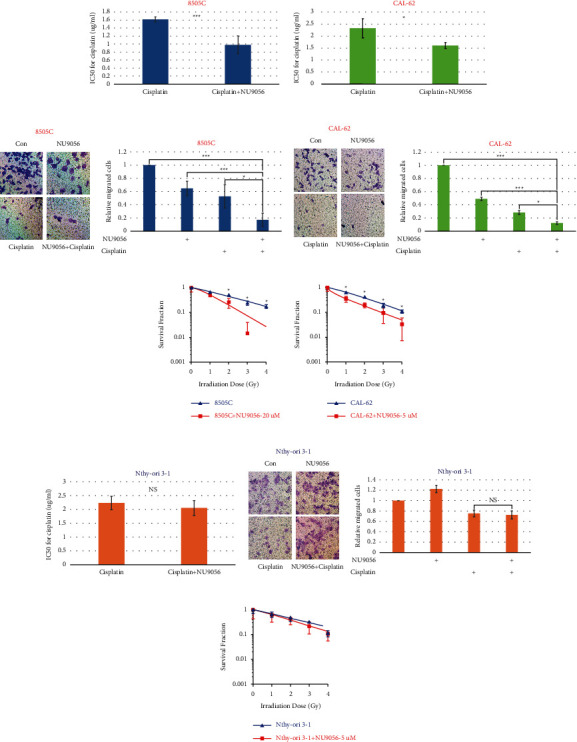
NU9056 increases radio- and chemosensitivities of human ATC cell 8505 C (A) C, (E), and CAL-62(B, (D) F), and Nthy-ori 3–1(G, (H) I) cells were treated with NU9056 or cisplatin and were further cultured in indicated time periods. Then, cell chemosensitivity was tested by CCK-8 (A) B, (G) or cell migration assay (C) D, (H). Next, cell radiosensitivity was tested by colony formation assay (E) F, (I). ^*∗*^*p* < 0.05 vs. “Ctrl” group, ^*∗∗∗*^*p* < 0.01 vs. “Ctrl” group.

The impact of NU9056 treatment on radiosensitivity and chemosensitivity of ATC cells was evaluated. The CCK-8 and Transwell assays both showed that NU9056 treatment increased the chemosensitivity of ATC cells to cisplatin (Figures 4(a)–4(d)). The radiosensitivity of ATC cells was also increased after NU9056 treatment (Figures 4(e) and 4(f)). Interestingly, NU9056 treatment showed no radio- or chemosensitization effects on normal thyroid cells with low KAT5 expression (Figures 4(g)–4(i)).

### 3.5. NU9056 Inhibits ATC Proliferation In Vivo

**Figure 5:**
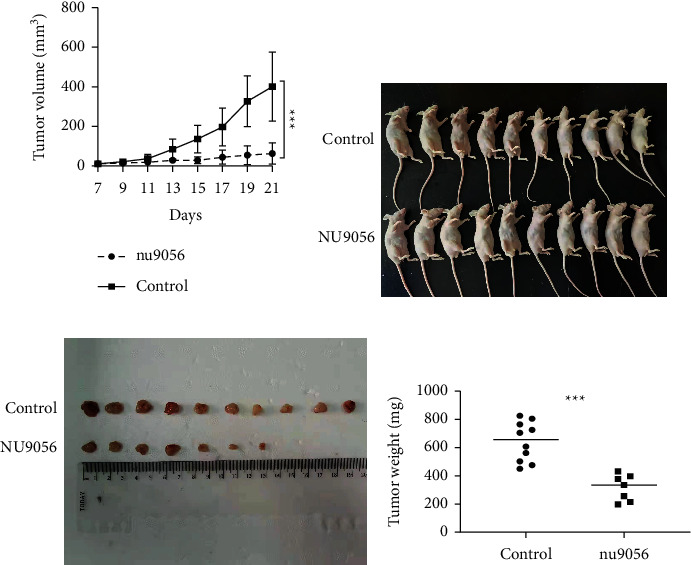
NU9056 inhibits ATC xenograft growth in mice. The SPF mice were injected ATC cells at the right flanks and were divided into the control or NU9056 group. The tumor growth curves were calculated (A). After 21 days, mice were killed and tumors were removed (B, C). Then, the tumor weights of each group were measured (D). *N* = 10 mice per group. ^*∗∗∗*^*p* < 0.01.

To investigate the potential antitumor activity of NU9056 on ATC *in vivo*, CAL-62 cells were injected into SPF mice to form subcutaneous xenografts. Then, these mice were randomly assigned to receive an intraperitoneal injection of either DMSO (as control) or NU9056. Tumor growth curves, in Figure 5(a), showed that xenografts derived from the NU9056 treatment group grew remarkedly slower. After 21 days, mice were killed and tumors were removed (Figures 5(b) and 5(c)). We found that the weights of tumors from the NU9056 treatment group were much lighter than those from the control group (Figure 5(d)).

### 3.6. NU9056 Inhibits miR-202-5p Expression in Human ATC Cells

**Figure 6:**
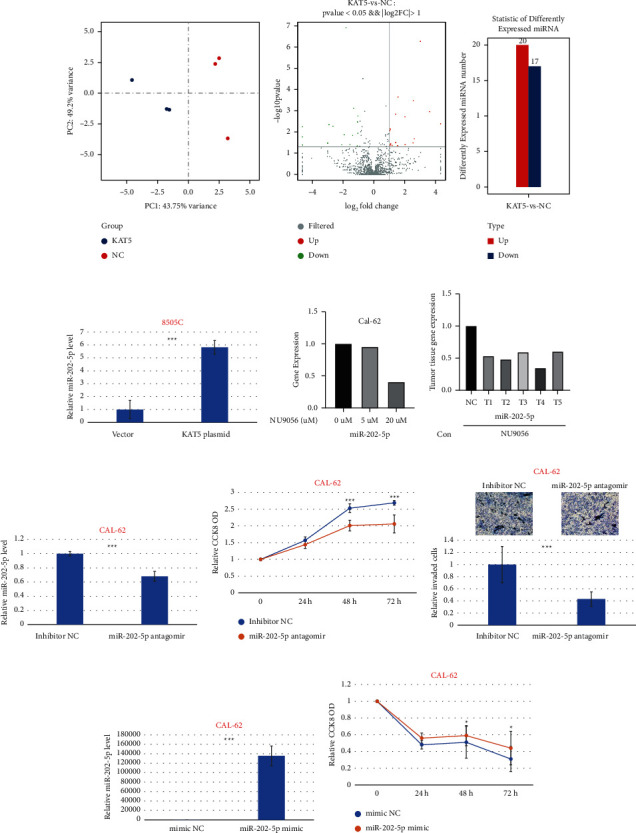
NU9056 inhibits miR-202-5p expression in human ATC cells. 8505C cells transiently transfected with either control or KAT5 plasmid, and microRNA sequencing was conducted (A-D). Cal-62 (E) cells were left untreated or treated with NU9056 (5–20uM), and the expression of miR-202-5p was detected by qRT-PCR (E). The SPF mice were injected with Cal-62 cells at the right flanks and were divided into control or NU9056 group. After 21 days, mice were killed and the expression of miR-202-5p of the tumor was detected by qRT-PCR (F). Cal-62 cells were transiently transfected with either control or miR-202-5p antagomir, and the knockdown effect was detected by RT-PCR (G). Then, cells were left untreated or treated with NU9056, and cell viability (H) and invasion ability (I) were tested by the appropriate assay. Cal-62 cells were transiently transfected with either control or miR-202-5p mimic, and the transfection effect was detected by RT-PCR (J). Then, cells were treated with NU9056, and cell viability (K) was tested by the appropriate assay. ^*∗*^*p* < 0.05 vs. “Ctrl” group, ^*∗∗∗*^*p* < 0.01vs. “Ctrl” group.

To identify the potential downstream miRNAs of KAT5 and NU9056 treatment, transfection of empty vector or KAT5 plasmid into 8505C cells was conducted. Then, miRNAs sequencing was conducted. We identified 37 miRNAs that showed upregulation or downregulation for at least twofold after overexpression of KAT5 in ATC cells (Figures 6(a)-6(c), Supplementary Table 1). Among them, the level of miR-202-5p dramatically increased, and this finding was further confirmed by qRT-PCR (Figure 6(d)). Data from qRT-PCR also consistently showed that inhibition of KAT5 by NU9056 significantly decreased the level of miR-202-5p (Figure 6(e)). Meanwhile, in animal study NU9056 also significantly decreased the level of miR-202-5p in CAL-62 xenograft tissue (Figure 6(f)). To detect the function of miR-202-5p in ATC progression, CAL-62 cells were transfected with inhibitor NC or miR-202-5p antagomir (Figure 6(g)). We found that knockdown of miR-202-5p inhibited proliferation and invasion of ATC cells (Figures 6(h), 6(i)). Furthermore, CAL-62 cells were transfected with mimic NC or miR-202-5p mimic (Figure 6(j)), and our data showed that the inhibitory effect of NU9056 on cell proliferation was partially blocked by overexpression of miR-202-5p (Figure 6(k)).

### 3.7. NU9056 Inhibits miR-202-5p Expression via Transcription Factor c-Myc

**Figure 7:**
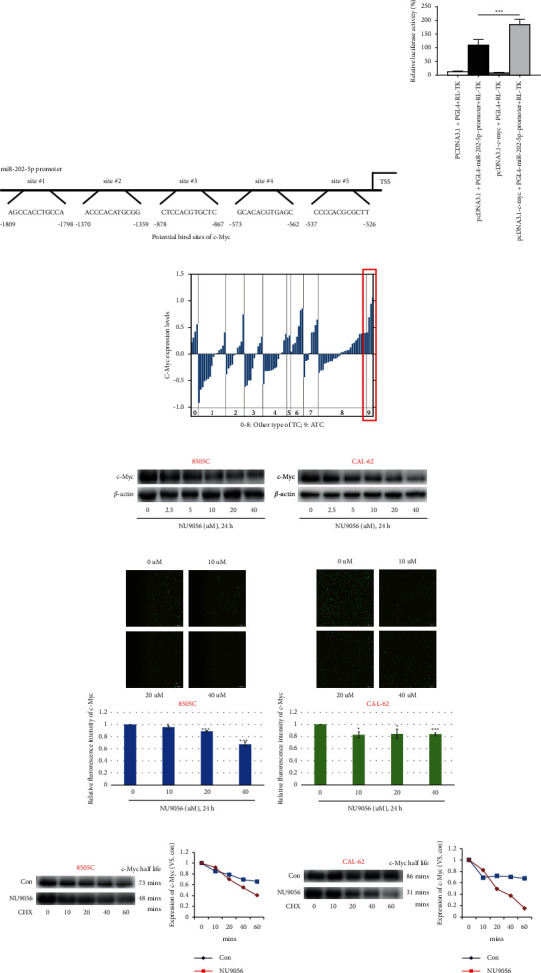
NU9056 inhibits miR-202-5p expression via transcription factor c-Myc. The possible transcription factors and the potential binding sites of miR-202-5p were predicted by JASPAR (A). 293T cells were transfected with pcDNA3.1 or pcDNA3.1-c-Myc together with PGL4+RL-TK or PGL4-miR-202-5p-promoter, and then, luciferase activity was measured (B). The c-Myc levels in different types of thyroid carcinoma in the Oncomine database (0: no value; 1: follicular variant thyroid gland papillary carcinoma; 2: tall cell variant thyroid gland papillary carcinoma; 3: thyroid gland follicular adenoma; 4: thyroid gland follicular carcinoma; 5: thyroid gland medullary carcinoma; 6: thyroid gland oncocytic adenoma; 7: thyroid gland oncocytic follicular carcinoma; 8: thyroid gland papillary carcinoma; and 9: anaplastic thyroid carcinoma) were analyzed (C). 8505C (D, E) and CAL-62 (D, F) cells were left untreated or treated with NU9056 (2.5–40uM), and then, the expression of c-Myc was detected by Western blot (D) or immunofluorescence (E, F). 8505C (G) and CAL-62 (H) cells were left untreated or treated with NU9056 (2.5–40uM), then, cells were treated with CHX (100 µg/mL) for indicated times, and c-Myc protein levels were analyzed by Western blotting. The half-life values were determined by densitometric quantitation using Image J (G, H).

To predict the transcription factors of miR-202-5p, the prediction program JASPAR was used. We found several potential c-Myc binding sites on the upstream promoter region of miR-202-5p, indicating that transcription factor c-Myc may regulate the level of miR-202-5p (Figure 7(a)). Next, dual-luciferase reporter assay showed that transfection of c-Myc significantly increased the luciferase activity of the PGL4-miR-202-5p promoter compared with the control (Figure 7(b)).

Our previous study proved that KAT5 promotes c-Myc expression via inhibition of its ubiquitin-mediated degradation in ATC cells [6]. We, therefore, sought to determine whether NU9056 treatment can affect c-Myc expression via regulation of ubiquitination. Oncomine database showed that c-Myc protein levels were most upregulated in ATC than in other types of thyroid carcinomas (Figure 7(c)). Next, Western blotting and immunofluorescence results both showed that NU9056 decreased the level of c-Myc expression in a dose-dependent way (Figures 7(d)–7(f)). Furthermore, after using cycloheximide to block protein synthesis, we found that NU9056 remarkably decreased the c-Myc half-life (Figures 7(g), 7(h)).

## 4. Discussion

Here, we examined the effects of NU9056, a highly selective KAT5 acetyltransferase inhibitor, on human ATC cells. Although tumors are typically considered to be a genetic disease, increasing evidence has shown that they initiated and progressed through both genetic and epigenetic alterations [16,17]. Therefore, novel drugs that target these epigenetic alterations could potentially provide a more tailored and specific response, which could have a significant clinical impact on tumor treatment [16,17]. Of these epigenetic alterations, reversible acetylation/deacetylation regulates several aspects of key cellular activities and has been the subject of much research [18,19]. Increasing evidence is being produced to suggest that the imbalance of acetylation/deacetylation leads to tumorigenesis and progression in various common cancers, including lung, breast, and esophageal cancer [16,20–24].

Our previous study reported that overexpression of KAT5 is associated with poorer survival and tumor metastasis in ATC and showed that KAT5 acetylates and stabilizes c-Myc by blocking proteasome [6]. KAT5 is the catalytic subunit of the evolutionarily conserved mammalian multi-subunit nucleosome acetyltransferase of the histone H4 complex, which mainly acetylates histone H4 and H2A. KAT5 is also recruited by several transcription factors and acetylates of several nonhistone substrates including androgen receptors, ATM, estrogen receptors, *β*-catenin, and E2F [7]. Moreover, KAT5 also plays important role in double-strand DNA break repair and apoptosis [7]. Therefore, KAT5 is involved in several pivotal cellular physiological and pathological processes, including carcinogenesis and tumor progression, and could be a novel potential therapeutic target for ATC treatment [6].

In this study, we showed that NU9056, a potent and highly selective KAT5 acetyltransferase inhibitor, inhibited survival, proliferation, migration, invasion, and tube formation and increased radiosensitivity and chemosensitivity in established human ATC lines (8505C and CAL-62) but showed no effect in human normal thyroid cells (Nthy-ori 3–1) and KAT5 downregulation ATC cell line. These results suggested that NU9056 selectively inhibited progression in KAT5 overexpressed ATC cells.

MiRNAs are single-stranded noncoding RNAs that include 21–25 nucleotides and play critical roles in several biological and pathological processes [13]. Dysregulation of miRNA plays a pivotal role in cancer formation and is commonly observed in several areas of human cancer studies including thyroid carcinoma, tumorigenesis, progression, therapy resistance, and prognosis predication [25–27]. The results of our miRNA sequencing and qRT-PCR experiments showed that upregulation of KAT5 increased levels of miR-202-5p, while inhibition of KAT5 by NU9056 decreased miR-202-5p expression. We also found that in ATC cells, knockdown of miR-202 suppressed progression, while forced expression of miR-202 partially blocked the inhibitory effect of NU9056. These results indicate that miR-202-5p could be the primary downstream target of KAT5. Previous studies have reported miR-202-5p to have tumor-suppressing qualities for many types of cancers including ovarian cancer, colorectal cancer, and bladder cancer [28,29]. Other study also showed that miR-202-5 is upregulated and associated with oncogene properties in breast cancer, lymphomagenesis, and osteosarcoma [30–33]. These findings bring into question whether the function of miR-202-5p as an oncogene/tumor suppressor is context dependent.

To investigate the mechanism of how KAT5 modulates miR-202-5p expression, we searched the JASPAR database. Based on the resultant prediction of the transcription factor and the binding sites, we suspect that c-Myc may be the transcription factor for miR-202-5p. The results of this study support our speculation that forced overexpression of c-Myc significantly increases the luciferase activity of the miR-202-5p promoter. Furthermore, we observed that the inhibitory effect of NU9056 on levels of miR-202-5p was partially rescued in c-Myc knockdown ATC cells. The powerful transcription factor c-Myc belongs to one of the most frequently overexpressed oncogenes, and its pivotal role in tumorigenesis and metastasis has been highly studied [11,12]. Interestingly, our previous study showed that KAT5-mediated acetylation stabilizes c-Myc via the inhibition of the ubiquitin-proteasome system [6]. The results of the current study are consistent with this, and in that we found that NU9056 inhibits c-Myc expression by decreasing protein stability. Taken together, these data indicate that KAT5 upregulates miR-202-5p expression at a transcriptional level through c-Myc.

**Figure 8:**
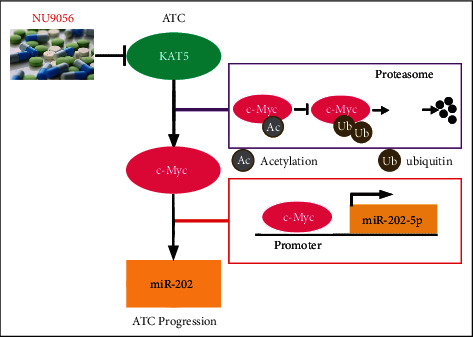
Schematic representation of KAT5 inhibitor NU9056 suppresses ATC progression via c-Myc/miR-202 pathway. In ATC cells, elevated KAT5 increases transcription factor c-Myc expression by promoting its protein stabilization. C-Myc then activates miR-202-5p transcription and mediates ATC progression. NU9056 inhibits ATC progression via the blockade of KAT5/c-Myc/miR-202 pathway.

Our results supported a model in which the aberrant overexpression of KAT5 stabilizes c-Myc protein, subsequently increases miR-202-5p level, and may cause ATC progression (Figure 8). Thus, inhibition of KAT5 by NU9056 could be a promising strategy for the clinical treatment of ATC (Figure 8). Meanwhile, the establishment of the KAT5/c-Myc/miR-202-5p axis will lead to a better understanding of molecular pathways involved in ATC.

## Data Availability

The data used to support the findings of this study are included within the article.

## Supplementary Materials

Supplementary Materials
*Supplementary Table 1*. Differently expressed miRNAs after overexpression of KAT5. MiRNA sequencing identified 37 miRNAs that showed upregulation or downregulation for at least twofold after overexpression of KAT5 in ATC cells.
