# Supplementary figures and images for: KAT5 Inhibitor NU9056 Suppresses Anaplastic Thyroid Carcinoma Progression through c-Myc/miR-202 Pathway
Source: Int J Endocrinol. 2022 Feb 11;2022:2014568. doi: 10.1155/2022/2014568 (PMC10279498; doi:10.1155/2022/2014568)

**Supplementary table 1.**


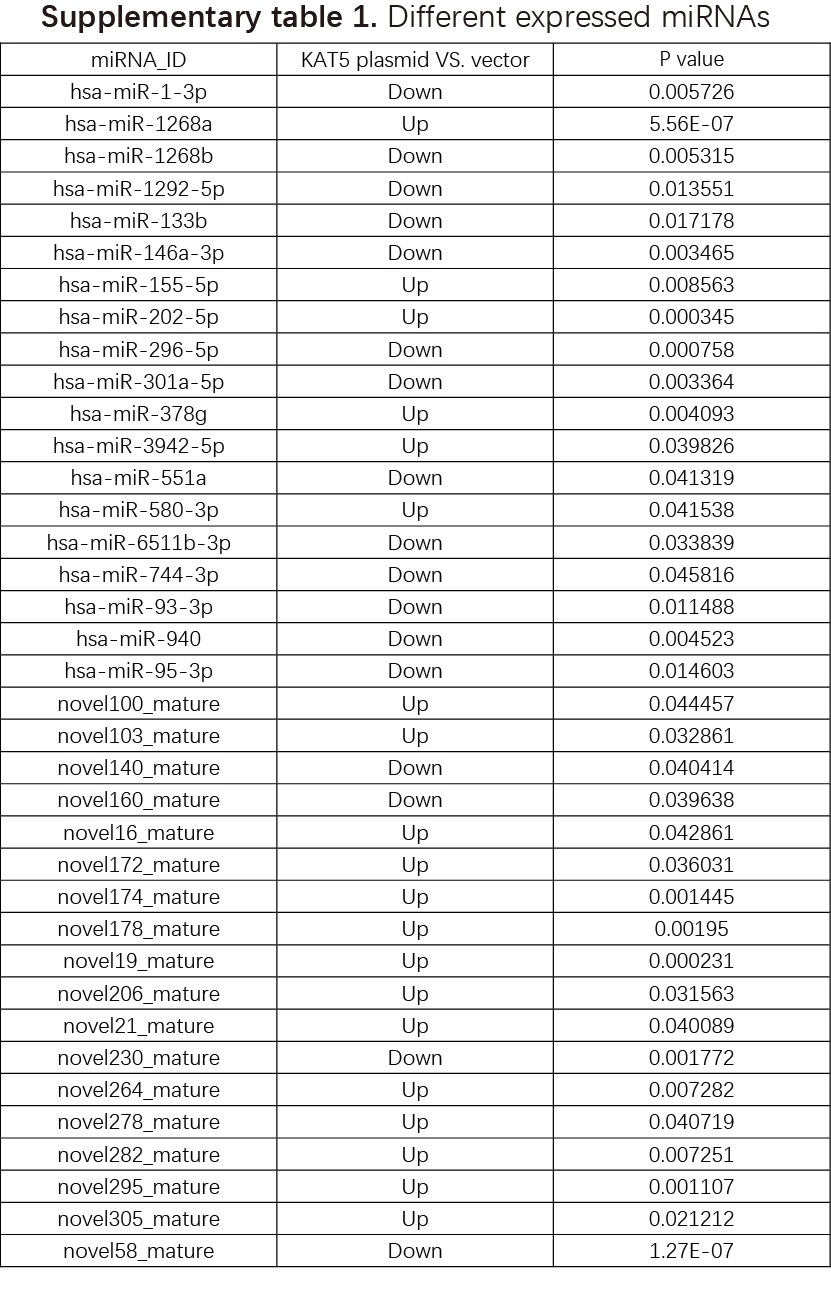

Supplement: Supplementary Materials — Supplementary Table 1. Differently expressed miRNAs after overexpression of KAT5. MiRNA sequencing identified 37 miRNAs that showed upregulation or downregulation for at least twofold after overexpression of KAT5 in ATC cells. [file 2014568.f1.doc]
